# Higher carotid-radial pulse wave velocity is associated with non-melancholic depressive symptoms in men – findings from Helsinki Birth Cohort Study

**DOI:** 10.1080/07853890.2021.1904277

**Published:** 2021-03-26

**Authors:** Mia D. Eriksson, Johan G. Eriksson, Hannu Kautiainen, Minna K. Salonen, Tuija M. Mikkola, Eero Kajantie, Niko Wasenius, Mikaela von Bonsdorff, Päivi Korhonen, Merja K. Laine

**Affiliations:** a Primary Health Care Unit, Helsinki University Hospital (HUS), Helsinki, Finland; b Folkhälsan Research Center, Helsinki, Finland; c Department of General Practice and Primary Health Care, University of Helsinki and Helsinki University Hospital, Helsinki, Finland; d Department of Obstetrics & Gynecology and Human Potential Translational Research Programme Yong Loo Lin School of Medicine, National University of Singapore, Singapore, Singapore; e Singapore Institute for Clinical Sciences (SICS), Agency for Science, Technology and Research (A*STAR), Singapore, Singapore; f Institute of Public Health and Clinical Nutrition, University of Eastern Finland, Kuopio, Finland; g Department of Public Health Solutions, Public Health Promotion Unit, Finnish Institute for Health and Welfare, Helsinki, Finland; h Faculty of Medicine, Clinicum, University of Helsinki, Helsinki, Finland; i PEDEGO Research Unit, MRC Oulu, Oulu University Hospital and University of Oulu, Oulu, Finland; j Department of Clinical and Molecular Medicine, Norwegian University of Science and Technology, Trondheim, Norway; k Gerontology Research Center and Faculty of Sport and Health Sciences, University of Jyväskylä, Jyväskylä, Finland; l Department of General Practice, Turku University Hospital and University of Turku, Turku, Finland

**Keywords:** Cohort study, comorbidity, depression, depressive disorder, pulse wave velocity

## Abstract

**Background:**

Depression and cardiovascular disease (CVD) are major causes of global disease burden that are interrelated through mostly unknown mechanisms. We studied the relationship of melancholic and non-melancholic depressive symptoms with arterial stiffness, an important underlying mechanism of CVD.

**Methods:**

The Helsinki Birth Cohort Study recruited 683 previously extensively phenotyped subjects for this sub-study. Cross-sectional data along with responses regarding depressive symptoms were obtained for each participant. For evaluation of depressive symptoms, the Beck Depression Inventory (BDI)and subscales were used to measure melancholic and non-melancholic depressive symptoms. Arterial stiffness was assessed as pulse wave velocity (PWV) that was measured between the carotid and radial artery, and carotid and femoral artery.

**Results:**

Of the participants, 532 scored <10 on the BDI and were classified as not having depressive symptoms. Of the 151 participants that scored ≥10 on the BDI, 122 were classified as having non-melancholic depressive symptoms and 29 as having melancholic depressive symptoms. Men had higher carotid-radial PWV (crPWV) values than women (*p* < .001). A positive relationship between BDI scores and crPWV (*p* < .001) was found in men. We also found higher crPWV in men with non-melancholic depressive symptoms compared to all others. No such differences were found in women.

**Discussion:**

Arterial stiffness has a relationship with depressive symptoms and subtypes of depressive symptoms, at least in men. There is a significant relationship between higher PWV and non-melancholic depressive symptoms in men. Due to the intricate nature of the disease causality or directionality is impossible to infer solely based on this study. Further studies into the subtypes of depressive symptoms may be of benefit to understanding depression.

**KEY MESSAGES**
- It is known that arterial stiffness contributes to cardiovascular disease, and is associated with depression.
- Higher Beck Depression Inventory scores are associated with higher carotid-radial pulse wave velocity in men.
- Non-melancholic depressive symptoms are associated with higher carotid-radial pulse wave velocity in men.

Over 322 million individuals worldwide are affected by depression, which is the leading cause of years lost to disability in the world [1] and an enormous contributor to the overall burden of disease [2]. The World Health Organization has predicted that by 2030 it will globally be the leading cause of burden of disease [3]. Depression has been shown to have been associated with a two-fold increased risk of death that cannot entirely be explained by behaviour or physical illness [4].

Depression is also strongly intertwined with physical health, including cardiovascular disease (CVD) [2]. Independent associations with depression for oxidative stress, increased inflammation, endothelial dysfunction, and arterial stiffening have been reported [5,6]. These same factors are also associated with CVD [5–7]. Recent research has shown an association between depressive symptoms and CVD even at depressive levels lower than indicative of depressive disorder [8]. This implies that there may be no lower limit of when depression begins to be associated with CVD [8]. It was also shown that this relationship between depressive symptoms and CVD is not dependent on cardiovascular risk factors [8].

The vascular hypothesis of depression is based on the concept that vascular disease can alter brain function by affecting perfusion and disrupting regional brain connectivity, which in turn can lead to clinical symptoms [9]. Arterial stiffness and increased flow pulsatility cause harm to the cerebral microvasculature [10–12]. Arterial pulsatility propagates directly from central vessels into cerebral vessels causing angiopathy [13]. Microvascular damages, especially in the frontal-subcortical mood regulatory regions, are believed to be one underlying cause of depression [11,14–20].

Arterial stiffness contributes greatly to the progression of CVD and reflects structural vessel wall changes leading to faster arterial wave propagation within the blood vessels [13]. Pulse wave velocity (PWV) is a method of assessing arterial stiffness in both clinical and subclinical vascular disease [21–26]. The measurement calculates the speed of the pulse wave in the aorta or peripheral arteries [23]. Prior studies have shown an association between depression or depressive symptoms and aortic stiffness in adults [5,27,28]. No such association has, however, been reported in older adults [27,29], nor in relation to melancholic or non-melancholic types of depression.

Prior research has indicated that melancholic and non-melancholic depression are associated with different underlying mechanisms [30,31]. Non-melancholic depression has been connected to metabolic and inflammatory dysregulations, whereas melancholic depression seems to be associated with an underlying hypothalamic-pituitary-adrenal (HPA)-axis dysregulation [30,31]. Dyslipidemia, high blood pressure and CVD are markers of metabolic dysregulation [30,31], leading us to hypothesize that non-melancholic depressive symptoms would have a stronger relationship with arterial stiffness than melancholic depressive symptoms.

The aim of this study was to assess arterial stiffness measured as PWV, and its relationship to depressive symptoms. We specifically focused on PWV differences between melancholic and non-melancholic depressive types. To the best of our knowledge, no studies have yet looked at PWV in relation to melancholic and non-melancholic depressive symptoms. Due to differing levels of cardiovascular risk factors and prevalence of depression between sexes we have also looked at any findings separately for men and women.

## Materials and methods

### Participants

The Helsinki Birth Cohort Study (HBCS) is composed of 13,345 men and women born between 1934 and 1944 at either Helsinki University Hospital or Helsinki City Maternity Hospital in Helsinki, Finland. Each subject attended child welfare clinics in Helsinki. The majority of subjects also attended school in Helsinki. Prior publications have detailed the records attained regarding birth, child welfare, and school health records [32,33].

By 1971 the members of the Finnish population were each given a unique identification number. From the original cohort, 8760 individuals (4630 men and 4130 women), all born at Helsinki University Hospital, were identified. In 2001–2004, 2902 subjects were identified for participation in further studies. These subjects were selected using random-number tables from those who were still alive and living in Finland. Out of the subjects identified 2003 individuals took part in the study. From this group of 2003, 1404 individuals were identified in 2011 as still being alive and living within 100 km of the study clinic in Helsinki. Of these subjects, 1094 participated in further clinical studies. In 2017–2018 these same criteria were used to identify subjects, of which 815 individuals participated in the study at hand. Of these, in 132 subjects PWV measurements were not obtained due to physical limitations of theparticipants or technical issues. These subjects were hence excluded from the study, and therefore 683 subjects had data available on the variables and were ultimately included in the current study.

### Ethics

The protocol (Dnro HUS/2020/2016) of the study was approved by the Coordinating Ethical Committee of the Hospital District of Helsinki and Uusimaa. Each subject gave written informed consent prior to participation. All study procedures followed the ethical guidelines set by the declaration of Helsinki.

### Depressive symptoms

The Beck Depression Inventory (BDI) was used to assess depressive symptoms among the subjects. The BDI is a 21-category self-reported questionnaire of behavioural manifestations of attitudes and symptoms specific to depression [34]. The range for the BDI is 0–63 points [35]. A cut-off of ≥10 total points was utilized to identify increased depressive symptoms. The BDI has been validated to screen for mild to severe clinical depression using the ≥10 point cut-off [35].

The subjects were classified as having either melancholic or non-melancholic depressive symptoms according to the presence or absence of melancholic symptoms (sadness, past failure, loss of pleasure, guilty feelings, punishment feelings, loss of interest, irritability, changes in sleep and appetite) as defined by the Diagnostic and Statistical Manual of Mental Disorders IV (DSM-IV) in a similar way as in prior publications [36–38]. The grouping into melancholic and non-melancholic depressive symptoms was determined by which summary score for symptoms outnumbered the other one, as described previously [36–38].

### Pulse wave velocity

PWV was measured using a Complior mechanotransducer sensor (ALAM Medical, France). All measurements were obtained in the same room by the same trained research nurse. Each subject rested for a minimum of 10 min before measurements were obtained. The subjects were supine and at rest, during which, two separate measurements of carotid-femoral pulse wave velocity (cfPWV) and carotid-radial pulse wave velocity (crPWV) were taken each. PWV was calculated as the distance between sites divided by the time between the upstroke of pressure waves. This was adjusted by a 0.8 scaling factor as recommended not to overestimate the true distance, and hence also PWV [39,40]. The PWV ratio was calculated as cfPWV divided by crPWV in a similar manner as in prior studies [41]

### Other measurements

Blood samples from each subject were drawn for laboratory assessment after an overnight fast. Measurements of glycated haemoglobin (HbA_1c_), high-sensitivity C-reactive protein (hs-CRP), and lipids were performed. A hexokinase method was utilized for the measurement of plasma glucose concentrations. Standard enzymatic methods were used to measure serum total cholesterol and triglyceride concentrations [42,43]. A Kawi stadiometer and a Seca Alpha 770 scale were used to measure height and weight, respectively. Body mass index (BMI) was calculated as the subject’s weight in kilograms divided by their height in metres squared (kg/m^2^).

Blood pressure was measured with the subject in a seated position. The recorded value was the mean of two successive readings from the right arm using a standard sphygmomanometer. Mean arterial pressure (MAP) was calculated as 1/3 (Systolic blood pressure) + 2/3 (Diastolic blood pressure).

A validated Kuopio Ischaemic Heart Disease Risk Factor Study 12‐month leisure‐time physical activity (LTPA) history questionnaire was utilized for assessment of LTPA [44]. Subjects were asked about their physical activity during the previous 12 months with regard to frequency (occasions per month), average duration, and intensity. A metabolic equivalent of task (MET)-value was assigned to each type of activity based on available databases (1 MET = 3.5 ml O_2_/kg/min) [45]. The total LTPA in MET‐hours (METh) is the MET values multiplied by the average duration and frequency of activity per week.

Questionnaires were utilized to gather information regarding alcohol consumption, smoking habits, health status, medication use, and socioeconomic variables (years of education, satisfaction with the financial situation, and cohabitation). Diseases diagnosed by a physician were inquired about, and the information was used to calculate the Charlson Comorbidity Index (CCI) [46]. Due to all subjects in the current study being within a narrow age range, age was not accounted for as a factor of CCI because our interest was in comorbidities without the effect of age.

### Statistical analysis

The characteristics are presented as means with standard deviations (SD) for continuous variables, and as frequencies with percentages for categorical variables. The depressive symptom groups were compared with the *t*-test for continuous variables, and Pearson’s chi-square test or Fisher’s exact test for categorical variables. A possible nonlinear relationship between BDI and the PWV was assessed by using 4-knot-restricted cubic spline regression. The length of the distribution of knots was located at the 5th, 35th, 65th, and 95th percentiles. For restricted cubic splines, also known as natural splines, knot locations are based on Harrell’s recommended percentiles [47]. The relationship between the type of depressive symptoms and sex in regard to PWV values was evaluated using a two-way analysis of covariance with age, CCI, MAP, smoking status, and years of education as covariates. In the case of violation of the assumptions (e.g. non-normality) for continuous variables, a bootstrap-type method was used. The normality of variables was evaluated graphically and by using the Shapiro–Wilk W test. The Stata 16.1, StataCorp LP (College Station, TX, USA) statistical package was used for the analysis.

## Results

Table 1 shows baseline characteristics of the 683 study subjects according to BDI score. Of the participants, 532 (78%) scored below 10 on the BDI indicating no depressive symptoms. Of the 151 (22%) participants scoring ≥10 on the BDI, indicating increased depressive symptoms, 105 (70%) were women, 122 (72% women) were classified as having non-melancholic depressive symptoms, and 29 (59% women) were classified as having melancholic depressive symptoms.

**Table 1.** Characteristics of subjects according to presence or absence of depressive symptoms as determined by BDI (<10 [no depressive symptoms] and ≥10 [presence of depressive symptoms]).

	BDI < 10 (*n* = 532)	BDI ≥ 10 (*n* = 151)	*p*-Value
Female, *n* (%)	273 (51)	105 (70)	<.001
Age (years), mean (SD)	76 (3)	77 (3)	<.001
Years of education, mean (SD)	13.1 (3.7)	12.5 (3.5)	.064
Cohabitating, *n* (%)	372 (70)	93 (62)	.045
Satisfied with financial situation, *n* (%)	326 (61)	66 (44)	<.001
Smoking, *n* (%)			.17
Never	256 (48)	86 (57)	
History of smoking	235 (44)	55 (36)	
Current smoker	38 (7)	10 (7)	
Alcohol use, *n* (%)			<.001
None	46 (9)	29 (19)	
Monthly	218 (41)	64 (42)	
Weekly	268 (50)	58 (38)	
Comorbidities			
Diabetes, *n* (%)	82 (15)	30 (20)	.19
CCI, mean (SD)	1.0 (1.6)	1.4 (1.7)	.027
Medication use, *n* (%)			
Use of antidepressants	22 (4)	28 (19)	<.001
Use of antipsychotics	3 (1)	1 (1)	.99
Use of anxiolytics	2 (0)	2 (1)	.21
Use of sleeping medication	10 (2)	3 (2)	.99
Use of lipid lowering medication	235 (44)	58 (38)	.21
Use of blood pressure medication	291 (55)	90 (60)	.28
Blood pressure (mmHg), mean (SD)			
Systolic	144 (20)	141 (18)	.070
Diastolic	78 (10)	78 (10)	.77
MAP	100 (12)	99 (12)	.26
LTPA (METh/week), mean (SD)	30.6 (23.8)	23.9 (22.0)	.002
BMI (kg/m^2^), mean (SD)	26.3 (4.0)	26.8 (4.0)	.15
Plasma glucose (mmol/l), mean (SD)	6.14 (0.91)	6.07 (0.85)	.38
HbA_1c_ (%), mean (SD)	5.57 (0.44)	5.58 (0.48)	.90
Cholesterol (mmol/l), mean (SD)	5.09 (1.11)	5.24 (1.20)	.16
LDL (mmol/l), mean (SD)	2.94 (0.94)	3.00 (1.01)	.44
HDL (mmol/l), mean (SD)	1.60 (0.37)	1.62 (0.42)	.58
Triglycerides (mmol/l), mean (SD)	1.23 (0.55)	1.36 (0.80)	.018
hs-CRP (mg/dl), mean (SD)	2.54 (5.20)	2.65 (5.40)	.78

BDI: Beck Depression Inventory; BMI: body mass index; CCI: Charlson Comorbidity Index; hs-CRP: high-sensitivity C-reactive protein; HbA_1c_: glycated haemoglobin; HDL: high density lipoprotein; LDL: low density lipoprotein; LTPA: leisure time physical activity; MAP: mean arterial pressure.

The subjects with depressive symptoms exercised less than the subjects without depressive symptoms when expressed as LTPA (*p* = .002). The subjects with depressive symptoms were also less likely to be cohabitating (*p* = .045), and less likely to be satisfied with their financial situation (*p* < .001) compared to their non-depressed counterparts. They also had higher triglyceride concentrations (*p* = .018), and CCI-scores (*p* = .027). There were no differences between the melancholic and non-melancholic groups in regard to these factors. As expected, those with depressive symptoms were more likely to use anti-depressant medications (*p* < .001). There was no significant difference in use of other medications between the groups. There was no significant difference in blood pressure, the prevalence of diabetes, blood glucose concentration, high-sensitivity C-reactive protein (hsCRP), BMI, or smoking status.

Mean cfPWV was 12.75 m/s (SD 4.25) in women, and 12.87 m/s (SD 4.53) in men (*p* = .71). The corresponding values for crPWV were 10.01 m/s (1.59) and 10.69 m/s (2.98), respectively (*p* < .001).

**Figure 1.**
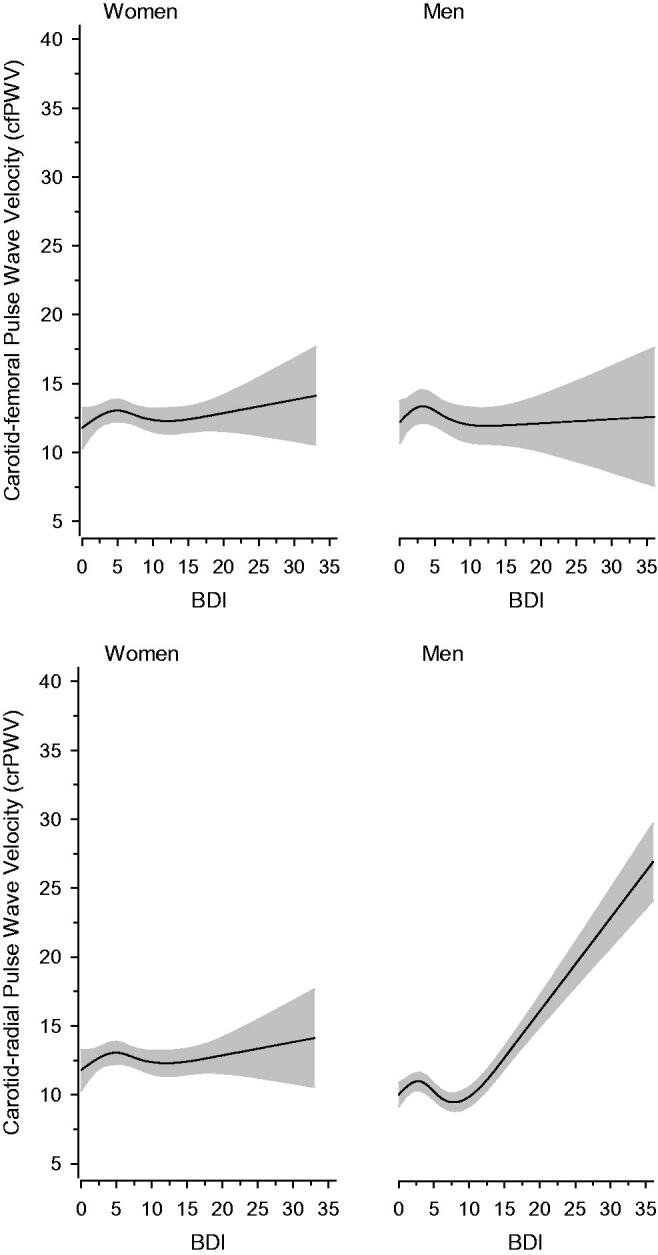
Relationships of pulse wave velocity (PWV) as the function of the BDI in men and women. The curves were derived from 4-knot-restricted cubic splines regression models. The models were adjusted for age, CCI, MAP, smoking status, and years of education. The grey areas represent the 95% confidence intervals.

Figure 1 shows the relationship between BDI scores and PWV according to sex. The values are adjusted for age, CCI, MAP, smoking status, and years of education. Among women, there was no relationship between BDI and cfPWV (*p* = .47) or crPWV (*p* = .50). This also applied to men and cfPWV (*p* = .30) in relation to BDI scores. There was, however, a significant relationship (*p* < .001) between BDI scores and crPWV in men.

**Figure 2.**
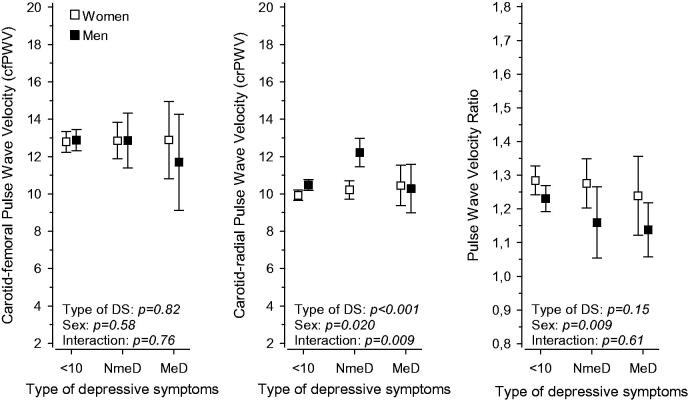
Pulse wave velocity (carotid-femoral and carotid-radial) in men and women based on type of depressive symptoms (measured by Beck Depression Inventory [BDI]); no depressive symptoms, non-melancholic depressive symptoms, or melancholic depressive symptoms. Adjusted for age, CCI, MAP, smoking status, and years of education. Ratio figure represents carotid-femoral pulse wave velocity divided by carotid-radial pulse wave velocity. DS: depressive symptoms; <10: <10 points on BDI, no depressive symptoms; MeD: melancholic depressive symptoms; NmeD: non-melancholic depressive symptoms.

Figure 2 shows the relationship between PWV and types of depressive symptoms separately for the sexes. This data was adjusted for age, CCI, MAP, smoking status, and years of education. For crPWV there was a significant difference (*p* < .001) between the subjects with melancholic and non-melancholic depressive symptoms, but no such difference was found with regard to cfPWV (*p* = .82). A difference between sexes was observed for crPWV (*p* = .020), but not for cfPWV (*p* = .58). No interaction was found between sex and type of depressive symptoms in relation to cfPWV (*p* = .76). For crPWV there was an interaction (*p* = .009) between sex and depressive types, with men in the non-melancholic depressive group showing the highest crPWV values. The ratio section in Figure 2 shows that there is a difference in PWV ratios between men and women (*p* = .009), but no difference between the types of depressive symptoms, and no interaction between depressive symptoms and sex in regard to the PWV ratio.

## Discussion

In accordance with previous studies, those participants who exhibited depressive symptoms were older, more likely women, and had a higher alcohol consumption. As we hypothesized, the results of this study support a stronger connection between vascular disease and non-melancholic depressive symptoms than melancholic ones. We believe this is due to the underlying causes of the depressive subtypes. As indicated in the introduction arterial stiffening falls under metabolic dysregulation, which is more closely associated with non-melancholic than melancholic depression [30,31]. We also found a difference between the sexes, and an interaction between sex and depressive categories (non-depressive, melancholic depressive symptoms, non-melancholic depressive symptoms). However, all of these findings were only true for crPWV, and not cfPWV. Prior research reported no association between cfPWV and depression or depressive symptoms, in either men or women above the age of 60 years [27], which is in line with our findings. This opens up the possibility that if our participants had been younger, we may have been able to see some type of relationship with cfPWV in addition to the one we found with crPWV.

In the present study, arterial stiffness was assessed by both carotid-femoral (aortic) and carotid-radial (brachial) PWV measurements. Our results show higher cfPWV than crPWV for both men and women, which is in agreement with central PWV increasing more and becoming higher than peripheral PWV with age [48]. The peripheral arteries of the human body are smaller in diameter and have more muscular and less elastic wall structures than the aorta [49,50]. There is evidence that the stiffness of medium-sized peripheral arteries is also modulated by endothelial function, the sympathetic nervous system, and the renin–angiotensin system [49,50]. Vasoconstriction and/or structural changes in the arterial wall alter the magnitude and timing of the reflected waves [51]. It has been shown that age has an effect on cfPWV, but no effect on crPWV in the general population [48,52], which may be one explanation for why no relationship is seen between depressive symptoms and cfPWV in the age group of the present study. Furthermore, others have reported that the association between CVD and depression is stronger in younger subjects than older ones [53].

The PWV ratio calculation is a representation of the arterial stiffness gradient [41]. The cfPWV/crPWV represents the aortic-brachial PWV ratio, which can be used as a measure of vascular aging [41]. With increasing age the arterial stiffness gradient reverses, enhancing microcirculation pulsatility and increasing the risk for organ damage [54]. When the gradient reverses the central PWV becomes greater than the peripheral PWV, and the PWV ratio will become >1 [48]. As our results indicate and are expected in this sample of older adults, both sexes and all groups have a PWV ratio >1 indicating a reversed gradient. In this study, the men had lower PWV ratios than women. Further, we observed sex differences in crPWV, but not in cfPWV. Based on this we can infer that the PWV ratio differences are likely caused by men having high crPWV readings rather than low cfPWV. The results in men are of interest because age should not increase crPWV [48], suggesting that the reason for these differences in the ratios between sexes is unlikely explained by age.

There is a known difference between men and women in PWV [27] and cardiovascular risk [55,56] in relation to depressive symptoms. This brings up the question of what causes these increases in PWV to only be noticeable in male subjects. Due to the age of the subjects at hand, any differences between the men and women are unlikely to have been caused by any hormonal differences between the sexes. Studies, such as the Maastricht study [27] and the Framingham study [52], have reported differences in PWV between men and women in younger age groups as well. This may indicate that any such differences may stem as far back as intrauterine effects on the cardiovascular system [57].

Recent studies have shown that the brain-gut-vascular axis may be a possible inflammatory connection between depression and arterial stiffness [58]. Psychoneuroimmune modulation may trigger arterial stiffness in patients with chronic inflammatory conditions [58,59], which could lead to the reasoning that depression may also cause similar inflammatory consequences. A few studies have been able to show that chronic inflammatory conditions are associated with higher crPWV [60–63]. In some cases, the combination of inflammation and aging seemed to be the culprit [62]. One study reported high crPWV in young women [60], and another study found a positive correlation between crPWV and the duration of inflammatory disease [63]. The takeaway may be that crPWV and cfPWV react differently in response to aging and inflammation, or the combination of the two. These findings may at least partly explain our results implying that stiffening of the peripheral, but not the central artery, is associated with increased depressive symptoms in men. It has been suggested that elevated smaller-artery PWV is a marker for early vascular disease having its origin in endothelial dysfunction whereas aortic stiffening is a marker for more advanced disease [64]. This may explain the observed sex difference since CVD develops later in women than in men.

It is also a possibility that the lower LTPA in participants with depressive symptoms, compared to their counterparts without depressive symptoms, higher triglycerides, and higher CCI-scores may play some part in arterial stiffening. When comparing the non-melancholic and melancholic groups in regard to these factors we observed no difference, but it is possible that there may be some differences that we are unable to see due to the small number of participants in the melancholic group. For better evaluation of this, a larger sample would be of benefit.

The strengths of the current study are the overall sample size and extensively phenotyped participants. The homogenous age range of the subjects helps control for effects of aging within the sample, which is of benefit in the study at hand. On the other hand, this may be seen as a limitation from the point of view of the generalizability of any findings from the current study. It is also likely that due to the nature of the sample we may have a survival bias where our sample may be healthier than the average person of the same age. We had to exclude some participants due to their inability to have their PWV measured, which suggests some of the less healthy participants were left out. Others with poor health may have already passed away or declined to participate due to their health status leaving this sample on average healthier. Other limitations of this study are the rather small size of each subgroup, a community-type sample of all-European-descent-subjects, and the cross-sectional nature of the study. The results here may hence not be generalizable to different populations. Due to the nature of the cross-sectional study, we cannot infer any directionality or causality, which limits interpretations of any results to simply observing relationships.

Further studies with larger subgroups and with subjects of different ages would be required to attain more information on these factors. Such studies would allow investigation of more detailed differences within each subgroup, potentially indicating factors not seen in the current study. A further comparison of relationships between PWV and depressive symptoms among subjects with and without prior CVD or vascular stenosis may advance our understanding of the pathophysiology of depressive symptoms. For example, simultaneous intra-arterial measurements and non-invasive PWV measurements could potentially be of interest in mapping out differences in results from cfPWV and crPWV. It would also be of great interest to study further the underlying mechanism of why our current findings are only significant for men.

The fact that depression is a heterogeneous disease whose subtypes are currently distinguished based on self-reported criteria instead of testable biomarkers can be problematic [65]. Melancholic and non-melancholic depression differ in both clinical presentation and pathophysiology [30,31] but continue to be viewed as one disease. Depression is treatable, but it is also increasing in prevalence, and up to half of the depressed individuals may not be receiving adequate treatment [65]. This offers the opportunity and needs for a better understanding of this complex disease and its subtypes. A better understanding of the pathophysiology of the depressive subtypes may enable us to eventually develop more precise and efficient treatments for depression. By analyzing differences between the depressive subtypes and their relationships to physiological mechanisms, such as PWV, we may gain more insight into why these subtypes behave so differently. The novel aspect of the current study is that, as far as we are aware, no other study has yet published results regarding PWV specifically analyzed by subtypes of depressive symptoms, and sex.

Due to the complex nature of depressive symptoms and their underlying causes, much research still lies ahead before we can conclusively determine a causal relationship. Based on the findings of this study it is likely that arterial stiffness is one of many factors affecting depression and depressive symptoms but that the overall pathophysiology is multifaceted and much more complicated. Even though there is a significant relationship between higher PWV and non-melancholic depressive symptoms in men, causality or directionality is impossible to infer solely based on this study.

## Data Availability

The data is available from the corresponding author upon reasonable request.
